# XRelevanceCAM: towards explainable tissue characterization with improved localisation of pathological structures in probe-based confocal laser endomicroscopy

**DOI:** 10.1007/s11548-024-03096-0

**Published:** 2024-03-27

**Authors:** Jianzhong You, Serine Ajlouni, Irini Kakaletri, Patra Charalampaki, Stamatia Giannarou

**Affiliations:** 1 Department of Computing, Imperial College London, Huxley Building, 180 Queen’s Gate, South Kensington, London, UK; 2 Medical Faculty, University Witten Herdecke, 58455 Witten, Germany; 3 Medical Faculty, Rheinische Friedrich Wilhelms University of Bonn, 53127 Bonn, Germany; 4 Department of Neurosurgery, University Witten Herdecke, 58455 Witten, Germany; 5 Department of Surgery and Cancer, Imperial College London, 413, 4th Floor, Bessemer Building, South Kensington Campus, London, UK

**Keywords:** Class Activation Map, Explainable Artificial Intelligence, Probe-based confocal laser endomicroscopy

## Abstract

**Purpose:**

Probe-based confocal laser endomicroscopy (pCLE) enables intraoperative tissue characterization with improved resection rates of brain tumours. Although a plethora of deep learning models have been developed for automating tissue characterization, their lack of transparency is a concern. To tackle this issue, techniques like Class Activation Map (CAM) and its variations highlight image regions related to model decisions. However, they often fall short of providing human-interpretable visual explanations for surgical decision support, primarily due to the shattered gradient problem or insufficient theoretical underpinning.

**Methods:**

In this paper, we introduce XRelevanceCAM, an explanation method rooted in a better backpropagation approach, incorporating sensitivity and conservation axioms. This enhanced method offers greater theoretical foundation and effectively mitigates the shattered gradient issue when compared to other CAM variants.

**Results:**

Qualitative and quantitative evaluations are based on ex vivo pCLE data of brain tumours. XRelevanceCAM effectively highlights clinically relevant areas that characterize the tissue type. Specifically, it yields a remarkable 56% improvement over our closest baseline, RelevanceCAM, in the network’s shallowest layer as measured by the mean Intersection over Union (mIoU) metric based on ground-truth annotations (from 18 to 28.07%). Furthermore, a 6% improvement in mIoU is observed when generating the final saliency map from all network layers.

**Conclusion:**

We introduce a new CAM variation, XRelevanceCAM, for precise identification of clinically important structures in pCLE data. This can aid introperative decision support in brain tumour resection surgery, as validated in our performance study.

## Introduction

Probe-based confocal laser endomicroscopy (pCLE) enables visualization of the tissue morphology at microscopic scale without changes in the surgical setting. Pilot studies verified that this technique can identify residual cancer tissue and improve resection rates of brain tumours. Automatic tissue characterization with pCLE would support the surgeon in establishing diagnosis as well as, guiding robot-assisted intervention procedures. Recently, Artificial Intelligence (AI) methods have been developed for this purpose. However, these high-capacity models face the significant drawback of lack of transparency in decision making, limiting their usage in interpretability-sensitive domains like AI-assisted diagnosis. Thus, the decision of a deep learning model supported by human-faithful visual explanations would facilitate tissue characterization. Particularly, visual explanations that better align with clinical knowledge enable the surgeon to place more trust in the model’s decision. To address this, Explainable Artificial Intelligence (XAI) has emerged and activation-driven methods like Class Activation Map (CAM) and its variants are popular XAI techniques that highlight salient image areas where the model has paid attention.

## Related work

Visual explanation is a convenient weakly supervised segmentation method in AI-assisted surgical interventions for decision support in tissue resection. Activation-driven method is a popular and computationally efficient class of explanation methods that aims to visualize the features learnt from a classification model, usually by applying a feature map weighting strategy in a layer of the model. The weighting factor formulation is different among the activation-driven methods. The earliest work in this category is the Class Activation Map (CAM) [1] and its popular variant Gradient-CAM (GradCAM) [2] that generalizes the former work by averaging the backpropagated gradient values (starting from the logit score of the target class) of a feature map in a layer as the weighting factor.

The axiom-based GradCAM (XGradCAM) [3] introduced two axioms to help impose theoretical supports in generating the weighting factor (the importance) on each feature map in a layer, namely, the conservation axiom and the sensitivity axiom. The conservation axiom is defined as $S_c(A^l) = \sum _{ij}\sum _{k} w_{lk}^c A_{ij}^{lk}$, where $w_{lk}^c$ is the weighting factor for the *k*th feature map in layer *l*, with respect to class *c*, $S_c(A^l)$ is the logit score of class *c* with activations in layer *l*, and $A_{ij}^{lk}$ is the activation value at map location (*i*, *j*). The aim of this axiom is to limit the non-explainable factors involved in the saliency map. The sensitivity axiom, on the other hand, encourages the weighting factor of a feature map in a layer to be the difference of the logit scores calculated before and after zeroing out the activation values of that feature map. Formally, it is expressed as $S_c(A^l) - S_c(A^l {\setminus } A^{lk}) = \sum _{ij}w_{lk}^cA^{lk}_{ij}$, where $S_c(A^l {\setminus } A^{lk})$ is the logit score of class *c* when zeroing out the *k*th feature map in layer *l*. Intuitively, the change in logit score when the feature map is removed is the empirical importance of that feature map.

Recently, RelevanceCAM [4] has been developed to mitigate the shattered gradient issue by using the Contrastive Layer-wise Relevance Propagation (CLRP) [5] paradigm which achieves remarkable weakly supervised segmentation results compared to other post hoc methods. This is particularly relevant in the medical domain where pixel annotations are scarce and localizing the semantic structure of tumours with models trained in a weakly supervised manner is highly desired. RelevanceCAM is using two propagation rules which have been proposed by the layer-wise relevance score propagation (LRP) method [6], namely, the LRP-$\epsilon$ rule and the LRP-$\alpha \beta$ rule. LRP-$\epsilon$ is defined as $R_i^{l_k} = \sum _{j}\frac{a_iw_{ij}}{\epsilon + \sum _{i}a_iw_{ij}}R_j^{l_{k+1}}$ and LRP-$\alpha \beta$ is defined as $R_i^{l_k} = \sum _{j}\left[ \alpha \frac{\max (0, a_iw_{ij})}{\epsilon + \sum _{i}\max (0, a_iw_{ij})} - \beta \frac{\min (0, a_iw_{ij})}{\epsilon + \sum _{i}\min (0, a_iw_{ij})}\right] R_j^{l_{k+1}}$, where $R_i^{l_k}$ is the spatial relevance score in layer *k*, $a_i$ is the spatial activation value, $w_{ij}$ is the weight connection between two neurons in layer *i* and layer *j*, and $\alpha$ and $\beta$ are hyperparameters. In CLRP, the above propagation rules run from the logit score of the target class after modifying the logit score of the non-target classes as $\frac{-L_\textrm{t}}{N-1}$ before the relevance backpropagation. $L_\textrm{t}$ is the logit score of the target class, and *N* is the total number of classes to be classified. Finally, the weighting factor used in RelevanceCAM is computed by simple averaging of all relevance scores in a feature map of a layer, computed by the aforementioned propagation rules.

**Fig. 1:**
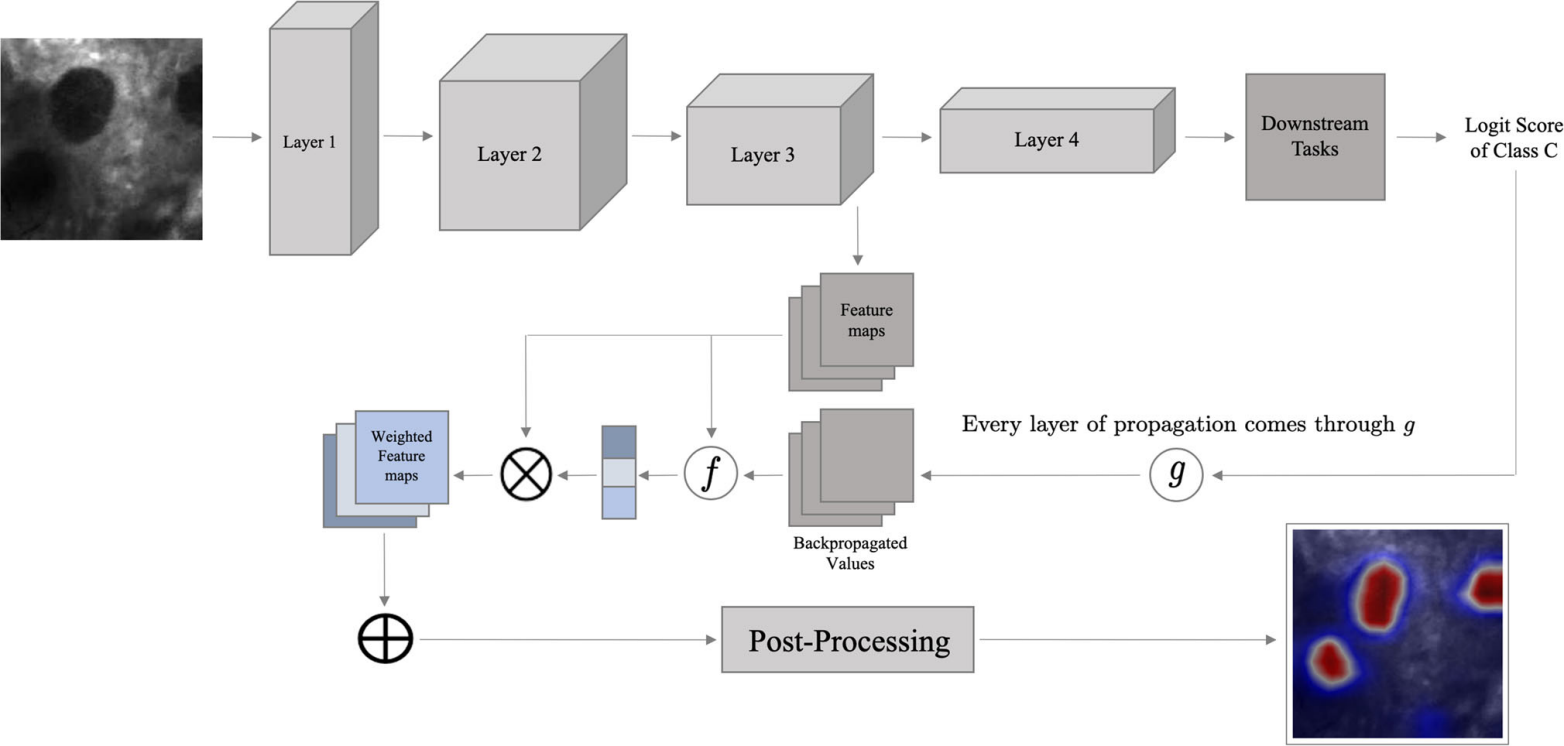
General pipeline of the weakly supervised segmentation process. *g* is novel propagation technique. Function *f* is a novel weighting strategy

Most of the CAM-based methods have been developed based on the vanilla gradient backpropagation, which is known to suffer from the shattered gradient problem that causes poor class-specified feature localization in the non-final layers [7]. However, features learnt by layers at different depth of a neural network architecture are significant in oncology. This is because spatial features learnt from the intermediate layers [8] and class discriminative features learnt from the final layers can help localize tumour regions semantically. The general relationship between the propagation-based methods, like guided propagation [9] and LRP, and the activation-driven methods is illustrated in Fig. 1.

### Contributions

When a model is trained with image labels, the state-of-the-art CAM-based methods either suffer from the shattered gradient problem or lack theoretical support to calculate the feature map attention factor in a layer. This causes sub-optimal localisation of pathological structures in AI-assisted decision making during intraoperative surgical interventions. In summary, the contributions of the paper are the following:

1. We propose a novel post hoc CAM-based method called XRelevanceCAM (Axiom-driven RelevanceCAM) that incorporates theoretical support into both the backpropagation technique and the feature map scoring mechanism.
2. With the generated saliency maps, the qualitative results show that our XRelevanceCAM is much more robust to the shattered gradient problem compared to RelevanceCAM. Also, it equips better semantic localisation ability of clinically relevant areas which characterize the tissue state compared to other state-of-the-art methods.
3. Through the performance evaluation study, we show that XRelevanceCAM outperforms the current state-of-the-art (SOTA) in terms of the mean Intersection over Union (IoU) metric in all layers of the deep learning model.
4. The robustness of the method is more significant when saliency maps from shallower layers are aggregated because it incorporates spatial information from these layers.

## Proposed methodology

### Image classification model

In our method, we adopt the pre-trained Selective Kernel (SK) of the ResNeXt-50 [10] model as our scale-invariant architecture to acquire multi-scale feature information. This is because semantic structures in medical images appear in multiple sizes. Ordinary deep learning models, like ResNeXt [11], use the same receptive field size to capture features which makes them less effective in recognizing image patterns that appear much larger or smaller than the receptive field size. The use of ResNeXt-50 as the backbone is for convenience only because SK-ResNeXt has a pre-trained copy available in the Timm open source library [12] for transfer learning. Our method is agnostic to the classification model but models that have innate ability to capture features at multiple scales are highly recommended for medical data.

### XRelevanceCAM for pathological structure localisation

#### Our method

Similar to other post hoc CAM-variant techniques, our framework consists of two components, namely, a chosen backpropagation method (*g* in Fig. 1 such as vanilla gradient or LRP) to obtain backpropagated values, and a novel feature map weighting method (*f* in Fig. 1) based on the propagated values in a layer. Although the CLRP propagation paradigm that RelevanceCAM has been using is known to be theoretically grounded [6] and mitigated the shattered gradient problem [7], its feature map weighting formulation lacks theoretical foundation (simple averaging of backpropagated values). In this paper, we use the same backpropagation setting as in RelevanceCAM to obtain the spatial relevance scores and propose a new feature map weighting strategy (Eq. 1) that satisfies the two axioms proposed by [3], namely, the sensitivity and conservation axiom, as much as possible. We form a new activation-driven saliency map method with the feature map weighting factor calculated as follows:

1 $$\begin{aligned} w_{lk}^c = \frac{1}{\sum _{ij} A_{ij}^{lk}}\sum _{ij}R_{ij}^{lk, c} \end{aligned}$$

where $R_{ij}^{lk,c}$ is the relevance score of a neuron obtained from the CLRP backpropagation with the modified class scores at location (*i*, *j*) of the *k*th feature map in layer *l* and propagated from class *c* and other notations are the same as before. In the analysis below, we represent $R_{ij}^{lk,c}$ by the function $R_{ij}^c(A^l; k)$. In the following sections, we provide the detailed derivation process behind the solution given in Eq. 1, by approximating the optimal solution of the two axioms *in tandem*

#### Problem formulation for the conservation axiom

Adopted from XGradCAM, we have the following optimization problem to find the optimal $w_{lk}^c$ that satisfy the conservation axiom [3]: $\underset{w_{lk}^c}{\textrm{argmin}} \left| S_c(A^l) - \sum _{ij}\sum _{k^{'}} w_{lk^{'}}^c A_{ij}^{lk^{'}} \right|$. By definition of the conservation axiom, the weighted sum of feature map activation values in each layer of the architecture should be equal to the logit score of the target class ($L_c = S_c(A^l)$ as a function of layer *l* activation $A^l$). However, in the CLRP propagation paradigm, each logit score of non-target class is modified to $-\frac{L_c}{N}$ (*N* is number of classes) and by the LRP-based conservation property, the sum of relevance scores in each layer for the target class is $L_c - (N-1)\frac{L_c}{N}$. Therefore, in our case, $S_c(A^l) = L_c - (N-1)\frac{L_c}{N} = \sum _k\sum _{ij}R_{ij}^c(A^l; k)$, with $N = 2$ and $R_{ij}^c(A^l; k)$ is the spatial relevance score as a function of the *k*th feature map in layer *l* activations. After rearranging the terms, we get $S_c(A^l) = L_c = \phi _c(A^l)\sum _k\sum _{ij}R_{ij}^c(A^l; k)$ and $\phi _c(A^l) = \left( (N-1) \frac{L_c}{N} + \sum _k\sum _{ij}R_{ij}^c(A^l; k) \right) \frac{1}{\sum _k\sum _{ij}R_{ij}^c(A^l; k)}$ for convenience. The final optimization problem for the conservation axiom becomes:

2 $$\begin{aligned} \underset{w_{lk}^c}{\textrm{argmin}} \left| \phi _c(A^l)\sum _{k^{'}}\sum _{ij}R_{ij}^c(A^l; k^{'}) - \sum _{k^{'}}\sum _{ij} w_{lk^{'}}^c A_{ij}^{lk^{'}} \right| \end{aligned}$$

For a particular $w_{lk}^c$ in layer *l*, we can solve the optimization problem by minimizing the $|\cdot |$ term:

$$\begin{aligned}&\phi _c(A^l)\sum _{k^{'}}\sum _{ij}R_{ij}^c(A^l; k) - \sum _{k^{'}}\sum _{ij} w_{lk}^c A_{ij}^{lk} = 0 \\&\quad \Rightarrow \phi _c(A^l)\sum _{ij}R_{ij}^c(A^l; k) = w_{lk}^c \sum _{ij}A^{lk}_{ij} \\&\quad \Rightarrow w_{lk}^c \\ {}&\quad = \frac{\phi _c(A^l)}{\sum _{ij} A_{ij}^{lk}}\sum _{ij}R_{ij}^c(A^l; k) \end{aligned}$$

Therefore, the optimal solution for the axiom-conservation property is $w_{lk}^c = \frac{\phi _c(A^l)}{\sum _{ij} A_{ij}^{lk}}\sum _{ij}R_{ij}^c(A^l; k)$. Our quantitative and qualitative evaluation shows that the $\phi _c(A^l)$ term does not have any effect on the method, and to simplify the expression, we set $\phi _c(A^l)=1$ and rewrite the optimal solution as Eq. 1.

#### Problem formulation for the sensitivity axiom

Adopted from XGradCAM, we have the following optimization problem to find the optimal $w_{lk}^c$ that satisfy the sensitivity axiom.

3 $$\begin{aligned} \underset{w_{lk}^c}{\textrm{argmin}} \sum _k \left| \left[ S_c(A^l) - S_c(A^l {\setminus } A^{lk}) \right] - \sum _{ij}w_{lk}^cA^{lk}_{ij} \right| \end{aligned}$$

Likewise, for each particular $w_{lk}^c$ in layer *l*, we find the solution by setting the $|\cdot |$ term to 0 so that Eq. 3 is minimized:

$$\begin{aligned}&\left[ \phi _c(A^l)\sum _{k^{'}}\sum _{ij}R_{ij}^c(A^l; k^{'}) - \phi _c(A^l {\setminus } A^{lk})\right. \\&\quad \left. \sum _{k^{'}:k^{'} \ne k}\sum _{ij}R_{ij}^c(A^l {\setminus } A^{lk}; k^{'}) \right] - \sum _{ij}w_{lk}^cA^{lk}_{ij} = 0\\&\quad \Rightarrow \left[ \rho (A^l; k) + \phi _c(A^l)\sum _{ij}R_{ij}^c(A^l; k) \right] = \sum _{ij}w_{lk}^cA^{lk}_{ij} \\&\quad \Rightarrow w_{lk}^c = \frac{\rho (A^l; k) + \phi _c(A^l)\sum _{ij}R_{ij}^c(A^l; k)}{\sum _{ij}A^{lk}_{ij}} \\&\quad \Rightarrow w_{lk}^c = \frac{\Psi (A^l; k)\phi _c(A^l)}{\sum _{ij}A^{lk}_{ij}}\sum _{ij}R_{ij}^c(A^l; k) \end{aligned}$$

where $R_{ij}^c(A^l {\setminus } A^{lk}; k^{'})$ is the recomputed spatial relevance score that satisfies the LRP-based conservation property [6] when $A^{lk} = 0$ in layer *l* and $\rho (A^l;k)$ and $\Psi (A^l; k)$ are defined as $\rho (A^l;k) = \sum _{k^{'}:k^{'} \ne k}\sum _{ij} \Big ( \phi _c(A^l)R_{ij}^c(A^l; k^{'}) - \phi _c(A^l {\setminus } A^{lk})R_{ij}^c(A^l {\setminus } A^{lk}; k^{'}) \Big )$ and $\Psi (A^l; k) = \frac{\rho (A^l; k) + \phi _c(A^l)\sum _{ij}R_{ij}^c(A^l; k)}{\phi _c(A^l)\sum _{ij}R_{ij}^c(A^l; k)}$. Therefore, the optimal solution for the sensitivity axiom is $w_{lk}^c = \frac{\Psi (A^l; k)\phi _c(A^l)}{\sum _{ij}A^{lk}_{ij}}\sum _{ij}R_{ij}^c(A^l; k)$. Note that the $\Psi (\cdot )$ term is hard to evaluate because it depends on the term $R_{ij}(A^l {\setminus } A^{lk}; \cdot )$ in the $\rho (\cdot )$ expression, which is the redistribution of relevance scores for layer *l* after the activation values in its *k*th feature map are swapped with 0. Notice that the optimal solution for the conservation and sensitivity axioms only differs in the $\Psi (\cdot )$ term which is hard to evaluate. Also, the common $\phi _c(A^l)$ term does not have any effect on the result. Therefore, to approximate both axioms in tandem, we set $\Psi (\cdot ) = 1$ and we arrive to the final estimated solution for both, given by Eq. 1.

### Layer-wise saliency maps aggregation

It is well known that shallow layers of a neural network tend to highlight the spatial details of an object but are not class discriminative, whereas upper layers exhibit the opposite case [13]. Therefore, we aggregate the saliency maps from all layers in hope that the result gets the best of both worlds. In our work, we generate one saliency map from each layer using XRelevanceCAM, average the saliency map values across all layers, and scale the averaged saliency map using the min-max normalization [14]. Our performance evaluations of the saliency maps aggregation show that the semantic localisation performance of tumour structure heavily depends on the explanation robustness of the shallow layers and the results show that our XRelevanceCAM is more reliable and captures more semantic details of the class discriminative features compared to other methods.

One limitation of this method is that the aggregation of saliency maps from shallower layers is subject to the representation quality (learnt feature quality) of the feature extractor. To fully unlock the potential of this technique, a powerful representation learning model such as SK-ResNeXt can be used because it can capture discriminative class features (e.g. psammoma bodies for the meningioma class) that are scale invariant. This is known to be a very useful property in training models targeted for medical images. On the other hand, if a less powerful model like Resnet50 is used, the final saliency map generated with layer aggregation may be less aligned with the clinical annotations. This is because, despite achieving very high accuracy in classification, Resnet50 makes predictions based on contextual information instead of discriminative class features.

## Experiments and results

### Data

*Database* Our dataset [15] consists of ex vivo pCLE videos from two types of brain tumours, namely, Meningioma and Glioblastoma (GBM). The data have been captured at a frame rate of about 15 frames per second. We have 16 patients in the GBM data and 18 patients in the Meningioma data, and all the data are grouped in the folder of their corresponding patients. Clinically relevant areas have been manually annotated by expert clinicians on the Meningioma data and correspond to psammoma bodies. Similar clinically salient areas cannot be defined for the GBM tumour class. We have a total of 12,392 images, with 5862 images in the Meningioma class and 6530 images in the GBM class. Hence, our dataset is approximately balanced. During the data splitting phrase, the splitting process is performed at the patient level. A random set of $80\%$ of all data is used for training (27 patients), a random set of $10\%$ of the data is used as validation set (three patients), and the rest serves as the test data (four patients) for performance evaluation of our proposed method.

*Data Pre-processing* At the pre-processing stage, we centre crop each frame to the size of the largest square space within the circle in each frame (230 pixels by 230 pixels) to remove black border areas and commercial logos. Subsequently, we take every other frame (even number indexed frames) in each video starting from the first frame because consecutive frames look very similar.

### Performance evaluation study

For performance evaluation, we use weakly supervised segmentation (WSS) and evaluate the mIoU between the segmented salient regions from the explanation map and the ground-truth manual annotations. The exact segmentation procedure from the explanation map refers to [3] and the task is performed on the annotated data only (Meningioma class). For all experiments, quantitative results of each evaluation metric are obtained using the correctly classified images and we assess the saliency maps from both qualitative and quantitative perspectives by comparing the performance relative to the most recent activation-driven methods.

### Model training and CAM implementation

We use the SK variant of ResNeXt_32x4d (SK-ResNeXt) [10] architecture as the backbone throughout all experiments unless explicitly specified. During the training phase, we use pre-trained weights to initialize the classification model and optimize the weights using cross entropy loss with image level labels. The learning rate begins with 0.001 and adjusts with the AdaMax [16] optimizer. The fine-turning process stops automatically after no consecutive improvement for ten epochs on the split-out validation data. Furthermore, random vertical flip, random horizontal flip, random rotation, and random colour contrast are the only data augmentations used during the fine-tuning phase to introduce variation in the data. The trained parameters are obtained from the Timm library [12] and model fine-tuning is done using the Pytorch framework [17] and Google Colab. Implementation of different CAM variants in the following experiments is obtained from the Torch-CAM library [18].

### Per-layer performance evaluation

**Fig. 2:**
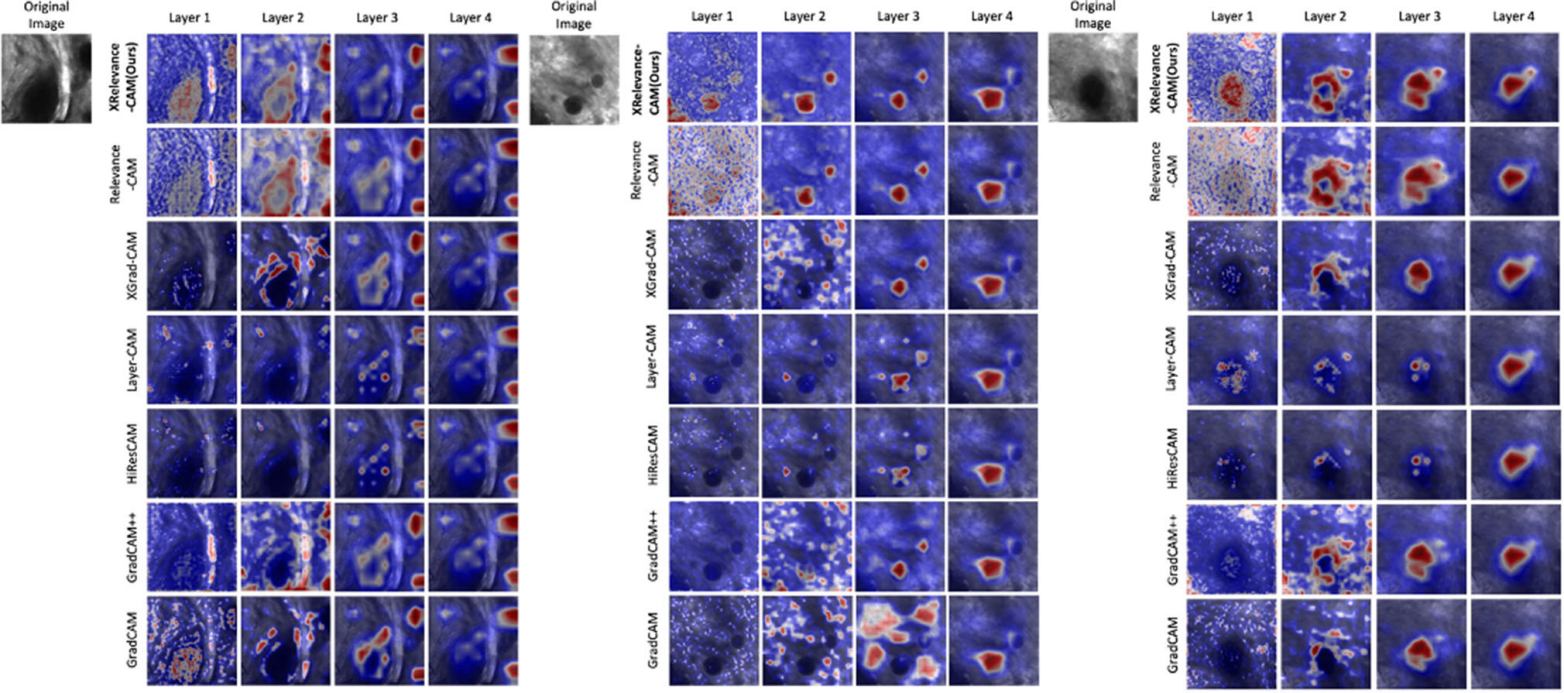
Comparison of various activation-driven methods for the sampled frames with SK-ResNeXt as the backbone. The first row contains the sampled test images and the saliency maps generated from our XRelevanceCAM. The black blobs are the target areas in the images

**Fig. 3:**
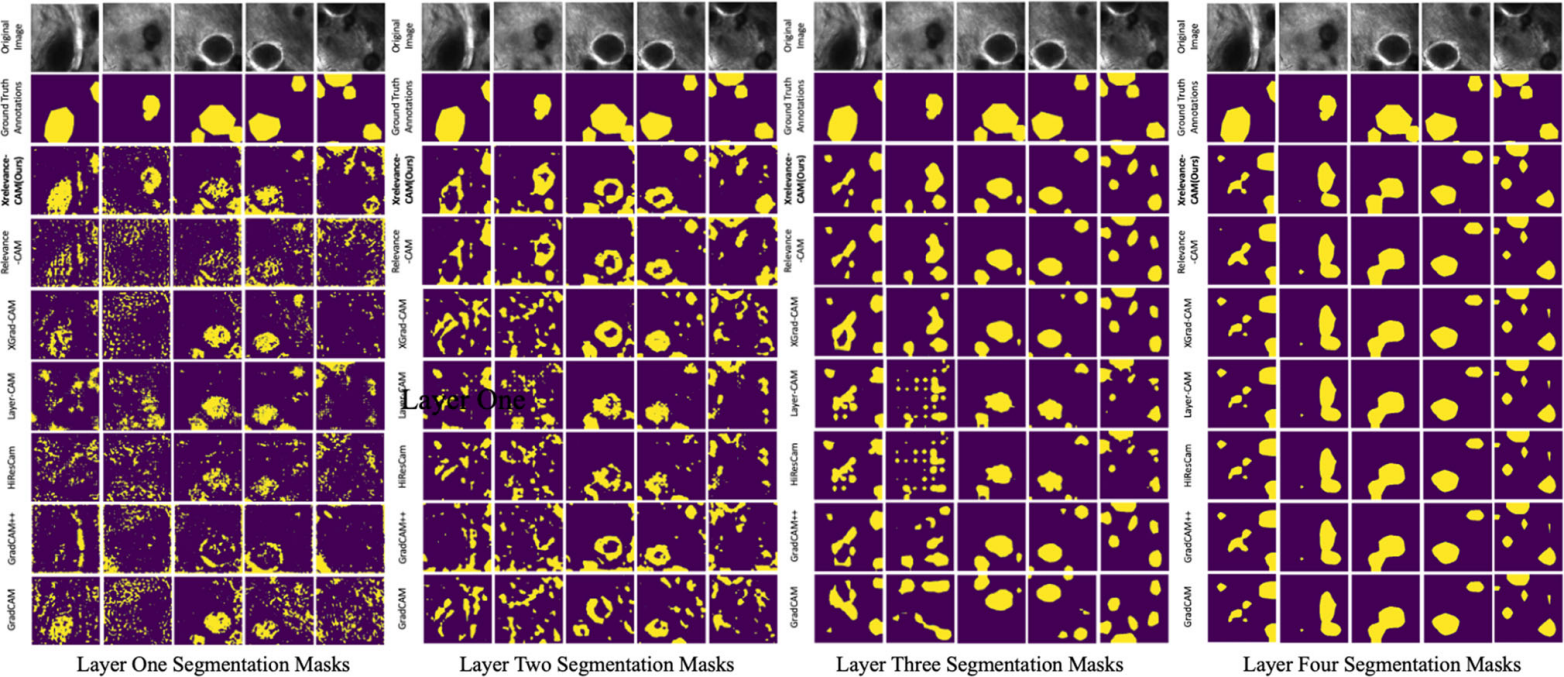
Saliency masks generated by each layer for various methods. The first row is the same set of sampled images for each layer. The second row shows the ground-truth masks of the relevant clinical structures. The third row shows the saliency masks generated from XRelevanceCAM

Table 1 shows the mIoU metric score for each layer, with respect to each CAM-variant. All methods have similar performance for the upper layers, and what differentiates the methods the most is the performance from the shallow layers. In particular, compared with our most competitive baseline RelevanceCAM, the marginal improvement reached as much as $10\%$ in layer one (a $56\%$ improvement), and the average per-layer performance of XRelevanceCAM exceeds $\approx 4\%$. Performance gain is much more noticeable compared to the widely used GradCAM and GradCAM++. Figure 2 shows the sample saliency maps for each layer generated by each CAM-based method to complement the quantitative findings. Figure 3 shows the saliency masks extracted from the saliency maps in each layer of the model, as well as the respective ground truths provided by the clinicians. In addition, the visualizations show that most of the methods are less robust in the shallow layers while XRelevanceCAM demonstrates a propensity for highlighting significantly fewer false positive tissue compared to the alternatives. This characteristic is of great significance in the context of AI-assisted tissue characterization during surgery, as the explanation that better aligns with the clinical knowledge earn more trust from (or give more confidence to) the surgeons.

**Table 1:** Per-layer IoU (%) performance in weakly supervised segmentation task, using SK-ResNeXt

Activation-driven methods	Layer 1	Layer 2	Layer 3	Layer 4	Average per-layer
GradCAM [2]	16.38	18.46	33.14	31.82	24.95
GradCAM++ [19]	8.44	20.23	35.01	32.15	23.96
XGradCAM [3]	14.40	24.77	**36**.**20**	31.82	26.80
HiResCAM [20]	15.06	21.93	30.56	31.82	24.84
LayerCAM [21]	22.19	27.50	32.0	31.70	28.34
RelavanceCAM [4]	18.0	30.37	33.42	31.0	28.19
**XRelevanceCAM (ours)**	**28**.**07**	**31**.**83**	35.11	**32**.**31**	**31**.**83**

Average Per-layer metric is obtained by averaging the numbers in its corresponding row. The best result is indicated in bold

### Evaluation on intermediate layers

Why bother to extract class relevant evidence from the intermediate layers could be a question that people raise. We adopt the argument from [4] where XRelevanceCAM only uses the relevance scores computed from CLRP propagation to find the per-channel weighting factor without modifying the spatial activation values of each feature map. As a result, deep neural network architectures possess an innate capability to identify class-specific features not only at deeper layers but also at the intermediate layers, going beyond just low-level semantic features such as edges. By incorporating a layer-wise aggregation mechanism, the resulting Class Activation Map effectively captures extensive semantic information, from all layers, pertaining to the tumour class.

### Evaluation of layer-wise saliency map aggregations

**Fig. 4:**
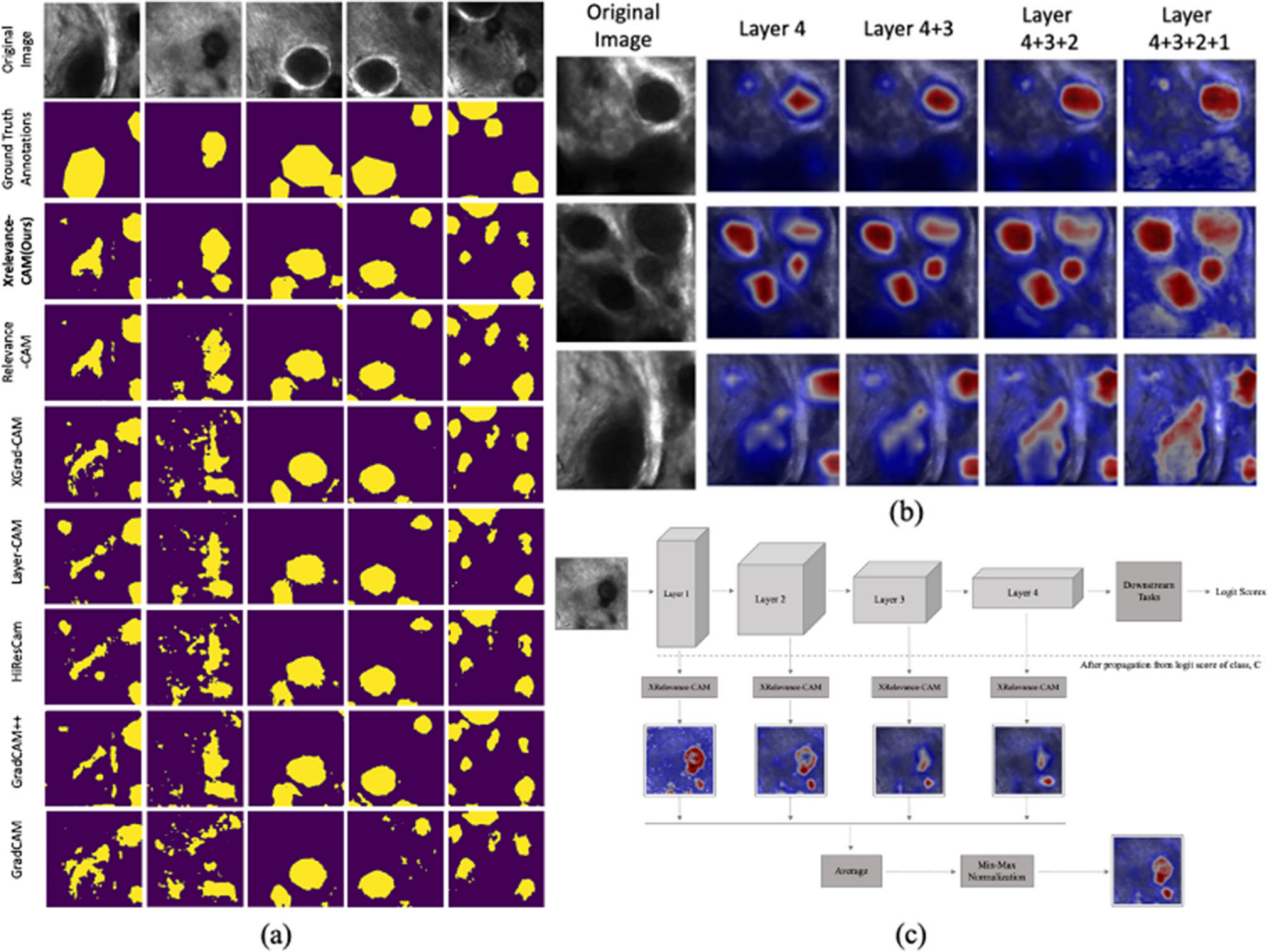
**a** Sample saliency masks generated with layer 4 + 3 + 2 + 1 for each CAM variants. **b** Sample saliency map of XRelevanceCAM when progressively aggregate an extra shallower layer. We see that more semantic details are captured with more layers involved in generating the saliency map **c** Workflow diagram of layer-wise saliency map aggregation of XRelevanceCAM. This also shows another example of gradually capturing the details of the psammonma bodies of the tissue (the black blobs)

We investigate the advantages of incorporating saliency maps from all layers in the context of the weakly supervised segmentation. Specifically, Table 2 presents much greater improvement in mIoU performance ($31.83\%$ vs $38.2\%$ in XRelevanceCAM) when shallower layers are included, using the SK-ResNeXt backbone. The incremental gain in performance decreases as we consider shallower layers. All compared methods exhibit inferior performance when layer one is taken into account, except our XRelevanceCAM. The saliency maps of shallow layers in Fig. 2 provide insight into the quantitative results where most methods give noisy explanation maps in layers one and two. Overall, the localisation performance of discriminative clinical structure is the best ($38.20\%$) when saliency maps of all layers are aggregated compared to other from a single layer, with the SK-ResNeXt backbone. On the other hand, as shown in Table 2, when the ResNet50 backbone is used the performance of XRelevanceCAM deteriorates if layer 2 or layer 1 is included. A similar performance drop is also observed for the other methods as well. This is likely attributed to the representation quality of the feature extractor as explained in Sect. 3.3. However, we should note that the partially/fully aggregated saliency maps using XRelevanceCAM still outperform other state-of-the-art CAM variants with the ResNet50 backbone. Sample saliency map results as well as saliency mask comparisons with the ground-truth masks are provided in Fig. 4.

**Table 2:** mIoU (%) performance in weakly supervised segmentation task with saliency maps aggregation, using ResNet50 [22] and Selective Kernel ResNeXt [10] as backbones

Activation-driven methods	Model	Layer 4	Layer 4 + 3	Layer 4 + 3 + 2	Layer 4 + 3 + 2 + 1
GradCAM [2]	SK-ResNeXt	31.82	33.86	32.73	31.74
	ResNet50	23.18	25.11	23.98	21.48
GradCAM++ [19]	SK-ResNeXt	32.15	34.87	35.32	33.79
	ResNet50	**24**.**47**	24.60	19.63	16.73
XGradCAM [3]	SK-ResNeXt	31.82	**34**.**99**	35.51	34.95
	ResNet50	23.18	27.24	25.92	24.78
HiResCAM [20]	SK-ResNeXt	31.82	32.77	33.33	32.61
	ResNet50	23.18	23.38	22.66	22.32
LayerCAM [21]	SK-ResNeXt	31.70	33.16	34.70	34.38
	ResNet50	23.15	24.01	22.54	21.64
RelevanceCAM [4]	SK-ResNeXt	31.0	33.13	35.79	35.93
	ResNet50	24.02	27.25	24.20	23.30
**XRelevanceCAM (ours)**	SK-ResNeXt	**32**.**31**	34.45	**37**.**04**	**38**.**20**
	ResNet50	23.68	**29**.**20**	**27**.**66**	**26**.**10**

Best result with respective to each model is highlighted in bold

### Sensitivity analysis using layer dropout

Previous experiments are evaluated based on a point estimation (one set of weights for the same model). However, the performance evaluation metric (mIoU) of explanation methods inherits the uncertainty from the stochasticity of model weights during optimisation. To better account for this uncertainty and inspired by the work from Gal et al. [23], we re-train a classification model with additional Dropout [24] layers and evaluate the explainability method with the dropout layers turned on. This setup simulates a collection of different neural network models which can be used to assess the average performance of an explainability method. In our experiment, each layer consists of multiple blocks of the same architecture. We modify the SK-ResNeXt model with one dropout layer after each layer with probability of dropout rate set to 0.1. The dropout mechanism is always turned on during the training and evaluation phase. In particular, the performance evaluation metric (mIoU) is obtained at the evaluation phase by passing each input ten times through the model and averaging the CAM result in each pass.

Table 3 shows the mIoU in each layer as well as that from aggregating saliency maps from all layers. With our XRelevanceCAM, we see that the average mIoU for layer one, layer two, and layer three exceeds the same metric for all other methods. The average mIoU performance from layer four is comparable to the other compared methods. Furthermore, the mIoU for layer aggregation also indicates that XRelevanceCAM outperforms the other methods. One remark is that when applying the dropout layer during the evaluation phase, the metric performances shown in Table 3 gets worse than usual. Therefore, the performance ranking between the compared methods is more important than the absolute mIoU value. To demonstrate the impact of using dropout during evaluation, we also include the mIoU results in Table 3 for the same model but discarding the stochasticity effect induced by the dropout after each layer.

**Table 3:** Sensitivity analysis using layer dropout for each CAM variants [10]

Activation-driven methods	Dropout [24]	Layer 1	Layer 2	Layer 3	Layer 4	Layer 4 + 3 + 2 + 1
GradCAM [2]	On	10.19	16.52	23.99	25.88	24.52
	Off	9.57	17.06	24.79	25.03	23.89
GradCAM++ [19]	On	9.97	11.11	26.42	**26**.**09**	25.59
	Off	9.23	12.14	27.03	25.29	25.54
XGradCAM [3]	On	12.15	17.55	24.81	25.87	26.58
	Off	11.57	18.73	25.05	25.03	25.82
HiResCAM [20]	On	9.14	15.12	21.68	25.87	25.57
	Off	9.51	15.28	22.41	26.00	25.21
LayerCAM [21]	On	9.22	14.85	22.41	25.99	24.49
	Off	9.72	16.05	22.86	25.13	24.51
RelevanceCAM [4]	On	17.87	22.08	28.08	25.98	28.57
	Off	12.69	26.71	35.37	31.90	35.20
**XRelevanceCAM (ours)**	On	**20**.**26**	**24**.**53**	**29**.**02**	25.96	**29**.**62**
	Off	**18**.**67**	**28**.**39**	**36**.**06**	**32**.**38**	**35**.**40**

Best result is highlighted in bold with respect to each dropout status in the modified SK-ResNeXt backbone

### Sanity check for XRelevanceCAM

**Fig. 5:**
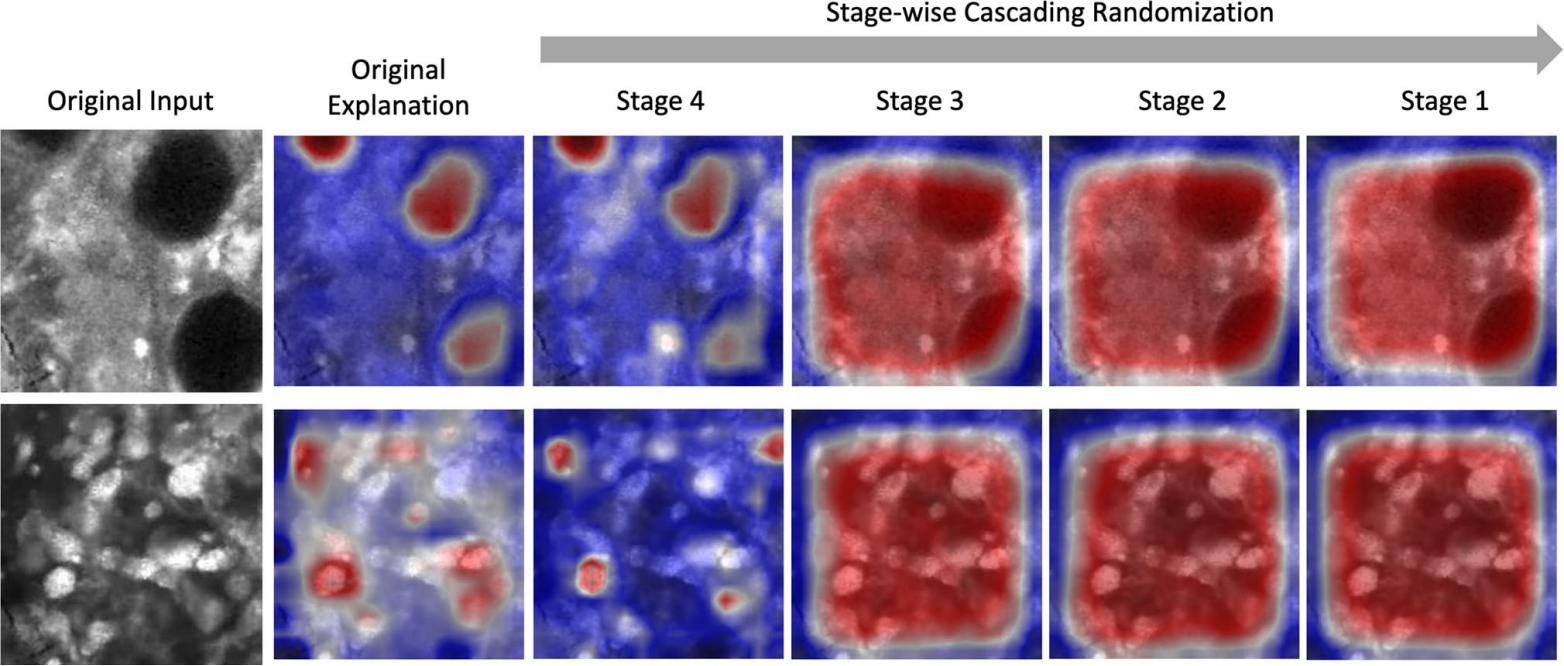
Visualization of the stage three in cascading layer-wise randomization on Resnet50 [22]. Top: sample Meningioma input. Bottom: sample Glioblastoma input

We follow the experimental procedure proposed by Adebayo et al.  [25] to evaluate the validity of our proposed XRelevanceCAM. We execute the cascading layer randomization task by progressively re-initializing the model with random weights stage-by-stage (layer-by-layer). Figure 5 shows the XRelevanceCAM visual results for layer three using the ResNet50 model and we see that the quality of the saliency map gradually deteriorates. According to [25], this demonstrates that XRelevanceCAM is a valid explanation method.

### Axiom evaluations

We adopt the same axiom analysis from [3] to verify the theory behind Eq. 1. Specifically, the performance for the sensitivity axiom is evaluated with the metric $\frac{1}{N} \sum _n^N \frac{\sum _k \left| S_c(A^l_n) - S_c(A^l_n {\setminus } A^{lk}_n) - \sum _{ij}w_{lk}^c R_{ij}(A^l_n; k) ) \right| }{\sum _k \left| S_c(A^l_n) - S_c(A^l_n {\setminus } A^{lk}_n) \right| }$ and the metric $\frac{1}{N} \sum _n^N \frac{\left| S_c(A^l_n) - \sum _k \sum _{ij}w_{lk}^cR_{ij}(A^l_n; k) \right| }{\left| S_c(A^l_n) \right| }$ is used to evaluate the conservation axiom, where $A_n^l$ is the activations of layer *l* for image *n*, $A_n^{lk}$ is the *k*th feature map activation in layer *l* for image *n*, $R_{ij}(\cdot )$ is spatial relevance score, and *N* is number of test images [3]. For fair comparisons, $S_c(\cdot )$ is the contrastive score (defined in Sect. 3.2) for RelevanceCAM and XRelevanceCAM, and set to the vanilla logit score for the rest of the methods. Evaluation results of the conservation axiom are reported in Table 4 and we see that our method has the best performance compared to the others. Regarding the sensitivity axiom, the results in Table 4 show that XRelevanceCAM outperforms RelevanceCAM and is comparable to LayerCAM. The above analysis indicates that our weighting strategy $w_{lk}^c = \frac{1}{\sum _{ij} A_{ij}^{lk}}\sum _{ij}R_{ij}^{lk, c}$ approximates both axioms well.

**Table 4:** Axiom evaluation (lower the better) in the split-out test data

Activation-driven methods	Axiom [3]	Layer 1	Layer 2	Layer 3	Layer 4	Average
GradCAM [2]	Conservation	1.26	0.88	0.61	0.001	0.689
	Sensitivity	1	0.999	0.999	0.999	0.999
GradCAM++ [19]	Conservation	460.11	628.16	105.31	5.484	299.77
	Sensitivity	2.912	2.09	0.925	**0**.**994**	1.73
XGradCAM [3]	Conservation	0.981	0.937	1.873	0.0018	0.948
	Sensitivity	0.998	0.994	0.994	0.999	0.996
HiResCAM [20]	Conservation	0.981	0.938	1.872	0.0018	0.948
	Sensitivity	0.998	0.994	0.994	0.999	0.995
LayerCAM [21]	Conservation	15.136	5.619	3.434	0.143	6.083
	Sensitivity	**0**.**904**	0.977	0.992	0.998	**0**.**968**
RelevanceCAM [4]	Conservation	0.56	0.12	0.13	1.30	0.528
	Sensitivity	1.01	0.93	0.82	2.40	1.29
**XRelevanceCAM (ours)**	Conservation	**0**.**02**	**0**.**01**	**0**.**0089**	**0**	**0**.**0097**
	Sensitivity	0.98	**0**.**91**	**0**.**79**	1.41	1.02

Best result with respective to each axiom is highlighted in bold

### XRelevanceCAM on ImageNet

**Fig. 6:**
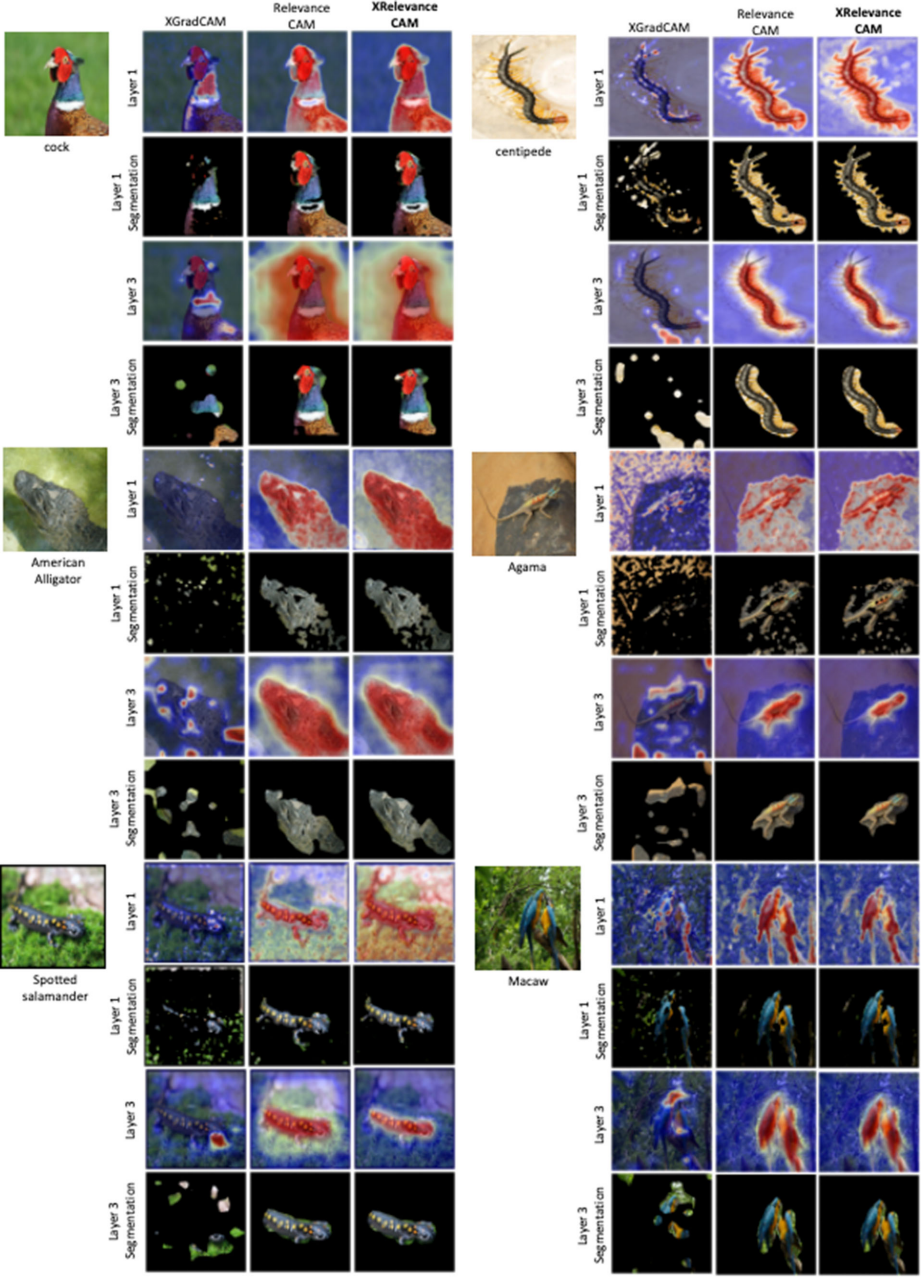
Each sampled image consists of four rows, including the saliency maps and the corresponding extracted saliency masks from layer one and layer three

To verify the generalisability of XRelevanceCAM on a different domain, we select ImageNet [26]. This is an alternative dataset to evaluate the target object localisation performance (the explainability) of our XRelevanceCAM because the characteristics of natural images are inherently different to medical images. Figure 6 shows sample images from ImageNet as well as, the saliency maps generated from layers one and three using the ResNet50 backbone. The figure also shows the saliency masks extracted from the corresponding saliency maps. We see that the qualitative results from XRelevanceCAM completely outperform XGradCAM and are on par with our main competitor, RelevanceCAM, in terms of target object localisation ability. Quantitative results are not included as ImageNet does not have ground-truth voxel annotations for evaluation. However, we do argue that from the qualitative results in Fig. 6, our XRelevanceCAM generalizes well to other datasets and domains.

## Conclusion

In this paper, we have introduced XRelevanceCAM that is more theoretically grounded and mitigates the shattered gradient problem that is shared by the most state-of-the-art CAM-based methods. The weakly supervised segmentation evaluation on pCLE data confirms that XRelevanceCAM successfully highlights the semantic structure of the tumours’ discriminative features, with the best outcome when saliency maps of all layers are combined. Extensive analysis verifies the potential of our proposed method to be used intraoperatively for AI-assisted tissue diagnosis during brain tumour resections and our XRelevanceCAM is generalizable to other datasets and domains.

Recently, XAI methods have received criticism due to their subjectivity [27] and inherently interpretable models like [28] gain popularity in high-stake decision making applications. In the case of AI-assisted surgery, extensive validation of XAI methods against ground-truth annotations defined by expert clinicians can provide confidence about the robustness of the applied XAI methods. A well-designed XAI method should be capable of revealing whether the model’s decisions are based on contextual features or on class-specific characteristics (e.g. presence of psammoma bodies on meningioma pCLE images). More importantly, surgeons should leverage XAI methods to enhance their understanding and trustworthiness of AI models, while remain responsible to make the final decision.

## Declarations

### Code availability

The code is available at the following link: https://github.com/CVRS-Hamlyn/XRelevance-CAM/tree/main.
